# Gut Microbiota as a Mediator of Essential and Toxic Effects of Zinc in the Intestines and Other Tissues

**DOI:** 10.3390/ijms222313074

**Published:** 2021-12-03

**Authors:** Anatoly V. Skalny, Michael Aschner, Xin Gen Lei, Viktor A. Gritsenko, Abel Santamaria, Svetlana I. Alekseenko, Nagaraja Tejo Prakash, Jung-Su Chang, Elena A. Sizova, Jane C. J. Chao, Jan Aaseth, Alexey A. Tinkov

**Affiliations:** 1 Laboratory of Molecular Dietetics, World-Class Research Center, Digital Biodesign and Personalized Healthcare, IM Sechenov First Moscow State Medical University (Sechenov University), 119146 Moscow, Russia; skalny3@microelements.ru (A.V.S.); michael.aschner@einsteinmed.org (M.A.); jaol-aas@online.no (J.A.); 2 Department of Bioelementology, K.G. Razumovsky Moscow State University of Technologies and Management, 109004 Moscow, Russia; 3 Department of Molecular Pharmacology, Albert Einstein College of Medicine, Bronx, NY 10461, USA; 4 Department of Animal Science, Cornell University, Ithaca, NY 14853, USA; xl20@cornell.edu; 5 Institute of Cellular and Intracellular Symbiosis, Russian Academy of Sciences, 460000 Orenburg, Russia; vag59@mail.ru; 6 Laboratorio de Aminoácidos Excitadores/Laboratorio de Neurofarmacología Molecular y Nanotecnología, Instituto Nacional de Neurología y Neurocirugía, Mexico City 14269, Mexico; absada@yahoo.com; 7 Saint-Petersburg Research Institute of Ear, Throat, Nose and Speech, 190013 St. Petersburg, Russia; svolga-lor@mail.ru; 8 Department of Otorhinolaryngology, I.I. Mechnikov North-Western State Medical University, 195067 St. Petersburg, Russia; 9 K.A. Raukhfus Children’s City Multidisciplinary Clinical Center for High Medical Technologies, 191036 St. Petersburg, Russia; 10 School of Energy and Environment, Thapar Institute Engineering and Technology, Patiala 147004, Punjab, India; ntejoprakash@thapar.edu; 11 School of Nutrition and Health Sciences, College of Nutrition, Taipei Medical University, Taipei 110, Taiwan; susanchang@tmu.edu.tw (J.-S.C.); chenjui@tmu.edu.tw (J.C.J.C.); 12 Graduate Institute of Metabolism and Obesity Sciences, College of Nutrition, Taipei Medical University, Taipei 110, Taiwan; 13 Federal Research Centre of Biological Systems and Agro-technologies of the Russian Academy of Sciences, 460000 Orenburg, Russia; sizova-l78@ya.ru; 14 Nutrition Research Center, Taipei Medical University Hospital, Taipei 110, Taiwan; 15 Research Department, Innlandet Hospital Trust, 2380 Brumunddal, Norway; 16 Laboratory of Ecobiomonitoring and Quality Control, Yaroslavl State University, Sovetskaya Str. 14, 150000 Yaroslavl, Russia

**Keywords:** zinc, gut microbiota, *Escherichia coli*, lipopolysaccharide, probiotic

## Abstract

The objective of the present study was to review the existing data on the association between Zn status and characteristics of gut microbiota in various organisms and the potential role of Zn-induced microbiota in modulating systemic effects. The existing data demonstrate a tight relationship between Zn metabolism and gut microbiota as demonstrated in Zn deficiency, supplementation, and toxicity studies. Generally, Zn was found to be a significant factor for gut bacteria biodiversity. The effects of physiological and nutritional Zn doses also result in improved gut wall integrity, thus contributing to reduced translocation of bacteria and gut microbiome metabolites into the systemic circulation. In contrast, Zn overexposure induced substantial alterations in gut microbiota. In parallel with intestinal effects, systemic effects of Zn-induced gut microbiota modulation may include systemic inflammation and acute pancreatitis, autism spectrum disorder and attention deficit hyperactivity disorder, as well as fetal alcohol syndrome and obesity. In view of both Zn and gut microbiota, as well as their interaction in the regulation of the physiological functions of the host organism, addressing these targets through the use of Zn-enriched probiotics may be considered an effective strategy for health management.

## 1. Introduction

Zinc is a IIB group metal essential for all forms of life [1]. The first studies on the biological essentiality of Zn^2+^ in fungi, plants, mammals, and humans originated more than a century ago [2]. The metal is involved in regulating the activity of >300 enzymes, mediating its role in a variety of biological processes. In the human organism, Zn plays a significant role in the development and functioning of the immune, endocrine, nervous, cardiovascular, and reproductive systems [3]. Due to the plethora of Zn-dependent processes, its deficiency is associated with multiple metabolic disorders, contributing to the pathogenesis of immune deficiency, neurodegeneration, diabetes mellitus, obesity, hypertension, and coronary heart disease to name but a few [2].

Competition between the host organism and pathogenic microflora due to the presence of high-affinity Zn transporters in the latter was shown to contribute to Zn in nutritional immunity [4]. Specifically, the host-specific mechanisms inducing limited Zn availability involve the modulation of Zn transporters [5], as well as the binding of Zn by calprotectin and other proteins including S100 proteins [6] and metallothionein [7]. In turn, bacterial cells have also evolved a broad spectrum of specific Zn transporters (e.g., ZnuABC) and Zn uptake regulators to promote the uptake of Zn^2+^ for their metabolic demands. Correspondingly, dysregulation of Zn^2+^ uptake due to ZnuA mutation results in altered growth and reduced virulence in bacteria [8].

Following the golden rule that “the dose makes the poison” (Paracelsus), excessive Zn levels may also be toxic for pathogenic bacteria. Particularly, Zn^2+^ may exert an inhibitory effect by interfering with Mn^2+^ metabolism [9], development of oxidative stress [10], and inhibition of biofilm formation [11].

In parallel to pathogenic microflora, zinc is also essential for intestinal commensal microflora inherent to the gut microbiome. The latter consists of more than 1000 bacterial species of various phyla with Bacteroidetes and Firmicutes being the predominant ones [12]. Recent findings have demonstrated that the gut microbiota is involved in the regulation of multiple functions of the host through the production of bioactive bacterial metabolites [13], thus being recognized as a novel human organ [14]. Specifically, gut microbiota was shown to play a significant role in the functioning of the immune [15], endocrine [16], reproductive [17], and other systems. The secretion of neuroactive metabolites underlies the functioning of the gut–brain axis and the role of gut microbiota in neuropsychiatric and neurodegenerative diseases [18].

The earliest indications on the impact of Zn on gut microbiota were obtained more than 30 years ago [19]. Since then, accumulating evidence has demonstrated an association between Zn deficiency and alterations in gut microbiota in chicks [20]. Multiple studies have assessed the impact of Zn supplementation on the gut microbiome in pigs with a special emphasis on diarrhea and growth [21]. Nonetheless, conclusions derived from animals, including laboratory rodents, cannot be confirmed in human studies due to insufficiency of the latter [22,23], although certain findings support the essential role of Zn for human microbiota [24,25]. Moreover, given the role of gut microbiota in human health and disease, it has been proposed that Zn-induced modulation of intestinal microflora and its metabolites may be involved in the physiological regulation of the host organism. In addition, the potential inconsistencies in the outcome of certain studies may be associated with the use of various Zn species that are known to possess different biological activities [26].

Therefore, the objective of the present study was to review the existing data on the association between Zn status and gut microbiota, as well as the role of this interplay in the physiological effects of Zn by addressing the following aspects:

1. The impact of Zn on characteristics of gut microbiota in various organisms.
  - Chicks
  - Piglets
  - Laboratory rodents
  - Humans
2. The influence of Zn on gut microbiota upon exposure to toxic and infectious agents.
3. The potential role of Zn-induced microbiota in modulating systemic effects with a focus on extraintestinal diseases.
4. Interactive effects of Zn and probiotics on gut microbiota.

## 2. Relationship between Zn Status and Gut Microbiota

### 2.1. Poultry

Zn deficiency was shown to be associated with altered gut microbiota in poultry [20]. Specifically, zinc deficiency in *Gallus gallus* was associated with a significant reduction in abundance of Firmicutes with a relative increase in Proteobacteria and Bacteroides. At the genus level, a significantly higher prevalence of unclassified *Ruminococcaceae* and *Enterobacteriaceae* and a reduced abundance of unclassified *Clostridiales* were observed upon Zn deficiency [20]. Feeding chicks a Zn-deficient diet also significantly reduced gut microbiota biodiversity with a significant decrease in the abundance of Firmicutes and an increase in Proteobacteria phyla. At the same time, at the genus level, the authors reported a significant increase in *Enterococcus*, *Enterobacteriaceae*, and *Ruminococcaceae* abundance, whereas *Peptostreptococcaceae* and *Clostridiales* were characterized by a significant decrease [27]. Correspondingly, in chicks fed Zn-fortified wheat, the abundance of *Ruminococcus* was considered the key genus associated with Zn status for discriminating between Zn deficiency and Zn repletion [28].

Contrary to Zn deficiency, supplementation of 15-day-old broilers with Zn bacitracin increased gut microbiota diversity, with a significant reduction in *Lactobacillus* and *Eubacterium* genus and an increase in the abundance of *Clostridiales* and *Faecalibacterium* [29]. In another study in broilers, Zn hydroxychloride supplementation significantly decreased total bacteria and Bacillus abundance, whereas *Lactobacillus* abundance was increased in parallel with cecal lactic acid production and up-regulation of intestinal tight junction proteins [30].

Zn supplementation was able to reduce the abundance of pathogenic bacteria in poultry. Supplementation of broilers with *Bacillus subtilis*-derived Zn nanoparticles significantly reduced ileal Coliform, *E. coli*, and *Salmonella* abundance, along with increased expression of tight junction proteins [31]. The competition for Zn binding between normal microbiota and *Campylobacter jejuni* in chicks was considered an antipathogenic mechanism [32].

Despite certain inconsistencies, which may be reflective of variations in dosing, treatment regimens, or chick characteristics, Zn appears to be beneficial for enhancing *Firmicutes* and decreasing the abundance of *E. coli* as well as certain other bacterial pathogens. Modulation of gut microbiota is also associated with improved gut wall integrity, thus contributing to gut health.

### 2.2. Pigs

In view of the significant hazards associated with post-weaning diarrhea in the pig industry [33], multiple studies have addressed the impact of Zn supplementation on the interaction between gut integrity and gut microbiota. Dietary exposure to coated ZnO in piglets resulted in a significant improvement in intestinal morphology and immunity, including increased villi length, elevated immunoglobulin A (IgA) levels, increased gene expression of IGF-1, occluding, zonula occludens 1, IL-10 and transforming growth factor β1 (TGF-β1), as well as reduced gut microbiota diversity. The latter was characterized by a decrease in the relative abundance of Lactobacillus and a non-linear response of *E. coli* numbers, which were increased at lower doses and down-regulated at higher concentrations of coated ZnO in diets [21]. Zn oxide supplementation in weanling piglets also resulted in increased jejunal mucosa TGF-β1 and IL-10 mRNA levels, whereas TNF-α and IFN-γ were decreased concomitant with the reduced abundance of Clostridium and *E. coli*, altogether resulting in the alleviation of postweanling diarrhea and growth performance [34]. ZnO was shown to reduce coliform bacteria abundance in piglets, leading to increased claudin-1 and zona occludens-1 gene expression, and these effects were strongly dependent on the source of Zn [35]. Taken together, these studies indicate that Zn supplementation diminished diarrhea in swine by improvement of intestinal integrity and immunity, down-regulation of inflammation, as well as modulation of gut microbiota.

It is notable that the effect of Zn on gut microbiota in weaned piglets is site-specific. In particular, ZnO nanoparticle (ZnONP) supplementation significantly reduced bacterial abundance and diversity in ileum with increases in *Streptococcus* and decreases in *Lactobacillus* numbers. In turn, cecal and colonic microflora biodiversity and abundance were increased, with a specific elevation in *Lactobacillus* numbers and a decrease in *Oscillospira* and *Prevotella* abundance. ZnONP—induced modulation of gut microbiome was associated with increased expression of tight junction and antioxidant proteins, as well as reduced IL-1β, TNF-α, and IFN-γ mRNA expression due to inhibition of NF-κB signaling, altogether resulting in lower incidence of diarrhea [36].

In agreement with the earlier studies, Starke et al. (2014) demonstrated that high dietary ZnO (2425 mg/kg) supplementation in weaned piglets reduced the abundance of Lactobacillus genus, and especially *L. acidophilus*, *L. mucosae*, and *L. amylovorus* throughout the full duration of the study (32–53 days), whereas *L. johnsonii* and *L. reuterii* responded weakly to dietary intervention. In addition, the relative number of *Enterobacteriacea* was found to be reduced at 35 days of treatment but not at later times. These findings demonstrate that the response of gut microbiota to ZnO exposure decreases significantly at older age [37]. High-dose dietary Zn oxide supplementation (3042 mg/kg) to piglets was shown to significantly modulate ileal bacterial diversity and relative abundance of Lactobacillus, Escherichia, as well as other minor species. Specifically, the majority of Enterobacteriaceae were characterized by a significant Zn-induced increase in relative abundance, whereas among bacterial species with relative abundance of >1%, Zn exposure resulted in a significant increase in *W. cibaria*, *W. confusa*, *Leuconostoc citreum*, and *S. equinus*. In contrast, the most abundant species L. reuteri decreased from 45% to 18% in response to Zn exposure [38]. Another study revealed a significant increase in intestinal microbiota richness and relative abundance of *Lachnospiraceae*, with a parallel decrease in *Ruminococcus flavefaciens* in response to coated nano zinc oxide supplementation [39].

In addition to modulation of microbiome richness and bacterial abundance, Zn was shown to prevent bacterial translocation from the gut to lymph nodes. Specifically, zinc-methionine supplementation in piglets during the nursing period significantly reduced the translocation of *E. coli* to small intestinal mesenteric lymph nodes [40]. Another study demonstrated a ZnO-induced reduction in anaerobic, and to a lesser extent lactic bacteria translocation to mesenteric lymph nodes in parallel with the elevation of intestinal IgA levels [41].

Zn was also shown to modulate microbial metabolite production through modulation of gut microbiota in pigs. Specifically, ZnO supplementation significantly increased total bacterial count with elevation of Enterobacteria and a decrease in *Clostridia* XIa cluster. The response of gut microbiota metabolites was shown to be non-linear with a significant increase in ileal volatile fatty acids, acetate, and butyrate at lower ZnO doses (50–150 mg/kg), and a subsequent decline to low levels at high ZnO concentrations. Only ammonia decreased with elevation of dietary ZnO doses [42]. In addition, an increase in microbial metabolites acetate, propionate, and butyrate was considered a marker of Zn sulfate supplementation in female pigs, and Zn-induced metabolic disturbances may significantly modulate the metabolic effects of heat shock exposure [43]. Correspondingly, a significant effect of ZnO supplementation on bacterial metabolites was observed, being characterized by a reduction in ammonia in the jejunum and colon, as well as lower lactate levels in the small intestine [37].

Therefore, the existing data clearly demonstrate the significant impact of Zn on porcine gut microbiota. Although the existing data are rather contradictory, being dependent on the mode of treatment and animal age, the most typical Zn supplementation-associated patterns may include increased bacterial richness, with a decrease in Enterobacteria and Lactobacillus abundance. Increased bacterial diversity and richness was also associated with elevated short-chain fatty acid levels, whereas lower lactate levels may correlate with reduced abundance of *Lactobacillus*. In addition to distinct changes in gut microflora, Zn supplementation in pigs was associated with improved gut integrity and consequently reduced bacterial and metabolite translocation to the systemic circulation.

### 2.3. Laboratory Rodents

A detailed study demonstrated that dietary Zn deficiency significantly affects gut microbiota in pregnant mice. Specifically, low dietary Zn significantly decreased the abundance of *Proteobacteria* and *Verrucomicrobia*, whereas *Actinobacteria*, *Bacteroidetes*, and Firmicutes phyla were increased. It is notable that the intake of Zn uptake inhibitors also resulted in the alteration of the gut microbiota, although the patterns were quite different, with a lack of significant changes in the abundance of *Verrucomicrobia* and *Actinobacteria*. The observed perturbations in gut microflora were associated with reduced Claudin3 protein levels in the gastrointestinal tract, altogether resulting in increased hepatic lipopolysaccharide (LPS) levels [44]. These findings are indicative of the essential role of Zn as a factor not only of impaired gut wall permeability but also gut microbiota. Being in agreement with the indications of the influence of Zn deficiency on gut microflora, a recent study demonstrated that Znt7 dysfunction also results in altered microbiota biodiversity, although the effects were sex-specific. Particularly, Znt7^+/−^ and Znt7^−/−^ genotypes were characterized by increased abundance of *Allobaculum* and unidentified members of the family Coriobacteriaceae in female, but not male, mice. It is also notable that these differences were associated with distinct patterns of mucin production, which were upregulated in male and down-regulated in female mice [45]. Concomitantly, another study demonstrated that dietary Zn deficiency did not cause substantial alterations in the gut microbiota in contrast to a protein-deficient diet [46].

In agreement with the studies demonstrating the essentiality of Zn for the gut microbiota, several studies have also shown that the modulation of intestinal microflora may mediate the beneficial effects of Zn. Although no significant alteration in bacterial phyla was observed in ZnCl_2_-supplemented mice, a significant increase in Zn-induced *Clostridiacea* abundance was observed in association with a significant improvement in gene expression responsible for metallothionein (MT) and mucin biosynthesis, and epithelial integrity, both in colon and intestine, as well as down-regulation of proinflammatory cytokine genes [47]. Concomitantly, the gut microflora response to Zn supplementation seemed to be age-dependent, being highly responsive to Zn status variability only in young animals, whereas at advanced ages no such effect was observed [48].

Despite the clearly demonstrated role of physiological doses of Zn in the adequate functioning of gastrointestinal and immune systems, high doses of Zn may cause adverse effects in the intestine [49]. Specifically, the exposure of newborn mice to high doses of Zn sulfate was shown to induce alterations of gut microflora biodiversity through an increase in *Pseudomonodales*, *Enterobacteriacae*, *Clostridiales*, *Bacteroides*, and *Campylobacter* abundance. Moreover, in the host, excessive Zn doses induced oxidative stress, reduced gut wall integrity, increased gut permeability, and affected intestinal gene expression with up-regulation of MT1, ALDH2, COX6b2, TMEM6, and CDK20, in parallel with CALU, ST3GAL4, CRTC2, SLC28A2 and COMMD1 down-regulation, thus affecting immune response, inflammation, and host–pathogen interaction. Altogether, these effects of Zn overload would be expected to contribute significantly to systemic inflammation and necrotizing enterocolitis [50]. In addition, chronic toxicity of ZnSO_4_ in mice (e.g., 250 mg/kg for 7 weeks) was characterized by reduced body and organ weight and increased AST activity was associated with a significant elevation in the relative abundance of *Enterobacteriaceae* without any impact on *Bifidobacteria* [51].

The existing data demonstrate that Zn deficiency is associated with profound alterations in gut microbiota composition that may contribute to proinflammatory conditions together with reduced gut wall integrity. However, Zn overload in laboratory rodents also promotes gut dysbiosis with a shift to Enterobacteriaceae and altered gut permeability, immunity, and inflammatory response.

### 2.4. Human

A limited number of studies have demonstrated the potential association between Zn status and human gut microbiota. Specifically, in vitro stimulators of the human colon demonstrated that ZnO nanoparticle exposure at high concentrations (50 mg/L) significantly reduced the abundance of gut microbiota as well as decreased bacterial biodiversity, SFCA production, and antibiotic resistance genes. The observed increase in relative abundance of Bacteroidetes was associated with a lower percentage of Firmicutes [22]. A preliminary study in Pakistani children demonstrated that formula-fed children with Zn deficiency are characterized by lower abundance of Escherichia, as well as decreased relative number of *Veillonella*, *Streptococcus*, *Bacteroides*, *Leuconostoc*, *Subdoligranulum*, *Megaspheare*, and *Clostridia*. However, correlation analysis did not reveal a strong association between serum Zn levels and intestinal bacteria [23].

In agreement with the essential role of Zn for gut microflora, patients with a pleiotropic missense variant of another Zn transporter, SLC39A8 (ZIP8), are also characterized by altered taxonomic characteristics of gut microbiota, including reduced abundance of *Anaerostipes*, *Coprococcus*, *Roseburia*, *Lachnospira*, SMB53, *Ruminococcaceae*, *Eubacterium*, *Dorea*, and *Bacteroides*. The patterns of gut microbiota observed in SLC39A8 Thr allele carriers shared several similarities with those shown in patients with Crohn’s disease and obesity [24]. At the same time, another study did not reveal any significant association between SLC39A8 missense variant and gut microbiota, although SLC39A8 [Thr]391 risk allele was genetically associated with Crohn’s disease [25].

Taken together, the existing findings from human studies demonstrate that Zn deficiency is associated with reduced gut microbiota biodiversity. However, no particular patterns could be ascertained given the paucity of existing limited data. Certain other studies involving human subjects demonstrated the potential involvement of the interplay between Zn and gut microbiota in other “extraintestinal” diseases and will be discussed in their respective sections.

### 2.5. Summary

Therefore, the existing data demonstrate that the effect of Zn on gut microbiota is species-specific (Table 1). Particularly, studies in chicks revealed a significant association between Zn sufficiency and Firmicutes, whereas the abundance of *Enterobacteriaceae* was decreased by Zn supplementation. In pigs, a similar trend for Zn-induced inhibition of *Enterobacteriaceae* colony growth was observed in parallel with a decrease in *Lactobacillus* abundance. At the same time, Zn was found to be a significant factor for gut bacteria biodiversity, consistent with findings in rodents and human subjects. Effects of physiological and nutritional Zn doses also result in improved gut wall integrity, thus contributing to reduced translocation of bacteria and gut microbiome metabolites into the systemic circulation.

In contrast, Zn overexposure also induced substantial alterations in gut microbiota with a shift to pathogenic strains of *E. coli* or other bacterial pathogens. Hypothetically, such an increase may be mediated by increased Zn levels exceeding the binding capacity, thus resulting in elevated “free” Zn available for bacterial pathogens, thus interrupting the mechanisms of nutritional immunity.

**Table 1:** A summary of studies demonstrating the impact of Zn on gut microbiota biodiversity and specific microbial taxa.

Species	Zn Form	Dose	Microbiota Biodiversity	Reduced Taxa	Increased Taxa	Ref.
Broilers	Zn bacitracin	50 ppm Zn	Increased	*Lactobacillus* *Eubacterium*	*Clostridiales* *Faecalibacterium*	[24]
Broilers	Zn hydroxychloride	20–100 mg Zn/kg Zn	Decreased	*Bacillus*	*Lactobacillus*	[25]
Piglets	Zn oxide	2250 mg Zn/kg	Decreased	*Lactobacillus* *E. coli* (at high doses)	*E. coli* (at low doses)	[21]
Piglets	Zn oxide	3042 mg Zn/kg (high dose)	Increased	*L. reuteri*	Enterobacteriaceae *W. cibaria* *W. confuse* *Leuconostoc citreum* *S. equinus*	[33]
Piglets	Zn oxide NPs	600–2000 mg Zn/kg	Decreased (ileum) Increased (cecum, colon)	*Lactobacillus* (ileum) *Oscillospira*, *Prevotella* (cecum, colon)	*Streptococcus* (ileum) *Lactobacillus* (cecum, colon)	[31]
Piglets	Coated nano ZnO	0.100 g Zn/kg diet	Increased	*R. flavefaciens*	Lachnospiraceae	[34]
Mice	Zn chloride	12–250 mg/kg b.w.	No effect	Lactobacillaceae Enterobacteriaceae	Clostridiacea	[42]
Mice	Zn sulfate	100 Zn µg/d (high dose)	Increased		*Pseudomonodales* Enterobacteriacae *Clostridiales* *Bacteroides* *Campylobacter*	[45]

The observed strain-specific response to Zn supplementation in bacteria may be mediated by differences in the amount of Zn^2+^ required to meet metabolic demands, as well as differences in tolerance to Zn [52].

In addition to the host species-specific response of gut microbiota to Zn, high heterogeneity of the findings may be associated with different biological effects of various chemical forms of the metal. Particularly, different impacts of zinc oxide, sulfate, or zinc oxide nanoparticles was observed in various species [53,54].

## 3. Zn and Microbiota upon Exposure to Toxic and Infectious Agents

Despite significant inconsistencies, existing evidence derived from studies on chicks, pigs, mice, and humans clearly indicate the essentiality of Zn for gut microbiota. In addition, Zn was shown to possess protective effects on gut microflora upon exposure to toxic agents, including pathogenic bacteria and physical or chemical stressors.

Specifically, exposure to doxorubicin, an anthracycline and antitumor antibiotic that affects cell growth through inhibition of DNA replication, induced a decline in Firmicutes and an increase in Bacteroidetes abundance. In turn, these alterations in gut microbiota biodiversity were shown to be ameliorated by Zn(II)-curcumin supplementation. At the genus level, Zn-curcumin supplementation also prevented a decrease in *Lachnospiraceae*, *Clostridium*_IV, *Clostridium*_XlVa, and *Roseburia*. These findings, together with improved gut wall integrity, mirror the observation of reduced fecal and plasma LPS concentrations [55]. Correspondingly, Zn(II)-curcumin complex was shown to ameliorate hepatocellular carcinoma-induced alterations in gut microflora by increasing the abundance of Firmicutes and decreasing Bacteroidetes, in addition to possessing anticancer effects itself and potentiating that of doxorubicin. The role of Zn-induced gut microbiota modulation in anticancer activity has also been supported by observations on the lack of such effects upon microbiome depletion [56]. In addition, it has been demonstrated that Zn deficiency alters gut microbiome and sensitizes it to As toxicity [57].

Along with the well-known mechanisms of nutritional immunity characterized by competition between host and pathogen for metals including Zn^2+^, Zn may also be a target for antagonism between commensal and pathogenic intestinal microflora. Specifically, it has been demonstrated that dual Zn-transporter system (ZnuABC and ZrgABCDE) in Vibrio cholerae mediate the advantage of the pathogen in competition for metal ions with gut microflora, thus being associated with *V. cholerae* growth and pathogenesis [58]. ZnuABC also significantly contributes to S. typhimurium, competing for Zn^2+^ ions with commensal bacteria, as well as assisting the pathogen to overcome calprotectin metal sequestration in the inflamed gut [58].

Being a target of commensal and pathogenic bacteria interaction, Zn was shown to modulate gut microbiota upon bacterial pathogen invasion. Specifically, in *S. typhimurium*-infected broiler chicks, supplementation with Zn significantly attenuated the hazardous effects of the infection through reducing apoptosis in intestinal cells, stimulating proliferation, increasing villi height, reducing Salmonella number, and reversing of *S. typhimurium*-induced reduction in gut microbiota diversity and *Lactobacillus* abundance [59]. Inhibition of bacterial translocation was shown to be associated with the maintenance of an adequate expression of intestinal tight junction proteins [60].

Concomitantly, it has been demonstrated that excessive dietary Zn supplementation significantly increased *C. difficile* toxin levels and aggravated clostridial infection [61], being associated with impairment in gut microbiota characterized by decreased *Turicibacter* and *Clostridium* genera, as well as increased *Enterococcus* and *Clostridium* XI genera abundance. In turn, binding Zn ions with calprotectin elicited a significant antibacterial effect [62]. These findings are indicative of the potential hazards of “free” Zn^2+^ upon overexposure, when the number of Zn ions exceed the Zn-binding capacity of the host organism. This hypothesis is indirectly supported by the observation of a significant improvement in symptoms and reduction in the risk of recurrence in Zn-deficient subjects with recurrent *C. difficile* infection following Zn supplementation [63].

Data from experiments on gut microbiota profiling demonstrated that commensal Enterobacteriaceae species, particularly *E. coli*, are one of the families most significantly affected by Zn supplementation. Accordingly, next, we discuss the interaction between Zn and *E. coli* with a special emphasis on pathogenic strains. Treatment with chitosan-chelated zinc attenuated the noted decrease in gut microbiota diversity in *E. coli*-challenged rats. In addition, Zn supplementation was associated with increased abundance of *Lactobacillus*, *Romboutsia*, *Clostridiales* (unclassified), and *Anaerotruncus*, whereas *Desulfovibrio*, *Peptococcus*, and particularly *E. coli* relative numbers were reduced. These changes were accompanied by a reduction in proinflammatory TNFα, IL-1β, IL-6 levels in parallel with up-regulation of IL-10 production. A tendency for improved total SFCA levels, and especially increased butyrate levels, was observed in Zn-supplemented animals challenged with *E. coli*. However, the lack of chitosan control group does not allow us to separate the effects of Zn from chitosan in the present study [64]. Correspondingly, dietary ZnO nanoparticles significantly reduced the intestinal *E. coli* population as well as increased villi height in the duodenum, jejunum, and ileum, resulting in improved immune response in weaned pigs [65].

A detailed analysis of 179 *Escherichia coli* genomes obtained from piglets after completion of a Zn oxide feeding trial demonstrated that genes and operons associated with virulence and bacteriocin production, as well as enterotoxigenic, enteropathogenic, and Shiga toxin-producing pathotypes were less abundant in high Zn-supplemented animals [66]. In enteropathogenic *Escherichia coli*, exposure to Zn was shown to reduce expression of virulence factors and reduced bacterial adhesion to the cells. Moreover, in rabbit ileum Zn was shown to ameliorate enteropathogenic *E. coli*-induced fluid secretion, thus being indicative of inhibitory effects of Zn on bacterial virulence factors [67]. It is also noteworthy that in parallel to reducing *E. coli* numbers, protective effects of Zn against gut leakage may involve the alleviation of *E. coli* alpha-hemolysin (HlyA)-induced alteration in tight junctions (claudins 4 and 5), focal leak formation, and cell exfoliation in piglet colonic tissue preparations [68].

It has also been demonstrated that *E. coli* may be less sensitive to dietary Zn as compared to beneficial bacterial strains, thus raising the risk of dysbiosis in response to inadequate Zn supplementation [69]. In particular, while *E. coli* growth and morphology were nearly insensitive to ZnO nanoparticle exposure, *L. acidophilus* and especially *B. animalis* growth was reduced in parallel with morphological deformation in response to increasing Zn exposure [52]. Moreover, high-dose ZnO feeding in piglets was associated with an approximately 15–20% increase in multidrug resistant *E. coli* as compared to controls [70].

Taken together, these data demonstrate that physiological Zn supplementation possesses protective effects on commensal gut microflora upon exposure to bacterial pathogens or xenobiotics, thus promoting the the health-supporting role of normal gut microbiota. However, excessive Zn exposure was shown to promote the growth and activity of bacterial pathogens due to the impairment of nutritional immunity mechanisms through exceeding the Zn-binding capacity of the host proteins.

## 4. Extraintestinal Effects in Models of Human Diseases

Although the majority of studies have linked the influence of Zn on gut microbiota with intestinal effects, such as gut wall permeability, inflammation, and intestinal metabolomics, a small number of studies aimed to assess its potential extraintestinal effects.

In agreement with the well-known anti-inflammatory effect of Zn [71], the earlier discussed studies demonstrated the role of microbiota-mediated decrease in LPS levels upon Zn exposure, which may, at least partially, underlie Zn’s modulatory effect on inflammation. In addition, it has been demonstrated that ZnSO_4_ reduced expression of constitutive (STAT1-induced) interferon-stimulated response (ISRE) genes and interferon regulatory factor (IRF) genes in intestinal epithelium, which was shown to be dependent on Zn-induced modulation of gut microbiota, altogether resulting in preventing excessive TNFα-dependent systemic inflammatory response [72]. In view of the role of systemic inflammation in the pathogenesis of various diseases [73], its modulation through Zn-induced changes in gut microbiota may be considered one of the mechanisms linking Zn metabolism with multiple pathologies.

As a particular case of the proposed mechanism, an earlier study demonstrated that Zn supplementation exhibits protective effects in a model of severe acute pancreatitis that appear to be at least partially dependent on the modulation of gut microbiota. Specifically, Zn sulfate supplementation in rats with pancreatitis significantly reduced endotoxic accumulation (LPS) and tissue IL-1β and TNFα expression, as well as attenuated pancreatitis-associated gut permeability and bacterial translocation to pancreas, liver, and mesenterial lymph nodes. The impact of Zn on gut microflora biodiversity was characterized by a reduction in Escherichia numbers, and an elevation of Bifidobacterium and Lactobacillus gene copy numbers in caecum [74]. Correspondingly, in patients with chronic pancreatitis characterized by high incidence (~40%) of small intestinal bacterial overgrowth, the latter was characterized by a significant negative correlation with serum Zn levels [75].

Modulation of gut microbiota was also considered a significant mediator of the regulatory role of Zn in immunity. Specifically, it has been demonstrated that Zn sulfate-supplemented mice are characterized by reduced gut microbiota biodiversity, as well as lower number and activity of T helper17 cells in murine small intestine. Moreover, transplantation of gut microflora to germ-free mice was associated with a significant influence on Th17 cells, indicative of the causal relationship between these processes [76].

However, Zn-mediated regulation of gut microflora is not linked only to immune and inflammatory pathologies. Specifically, recent findings also unravel the potential contribution of Zn in the modulation of the gut–brain axis in autism spectrum disorder and other neurodevelopmental disorders.

An earlier discussed study by Sauer and Grabrucker (2019) demonstrated that Zn deficiency-associated alterations to gut microbiota, increased gut permeability, and elevated systemic LPS levels are also associated with increased brain IL-6 levels and glial fibrillary acidic protein (GFAP) expression in the brain, thus being indicative of the role of altered gut microbiota, increased intestinal permeability, and endotoxinemia in neuroinflammation [44]. The authors also proposed that gut microbiota should be considered as the potential link between zinc deficiency and autism spectrum disorders [77]. Correspondingly, in an autism model of Shank3B ^-/-^ KO mice, Zn supplementation (150 ppm) was shown to revert alterations in fungal and bacterial diversity, modify expression of tight junction genes, as well as genes involved in immune diseases and energy metabolism [78]. In corroboration, a recent study demonstrated that ZnONP supplementation in children with autism spectrum disorder (ASD) was associated with significant improvement in intestinal bacterial biodiversity, ameliorated ASD-associated increases in Proteobacteria abundance, as well as a reduction in relative Firmicutes and Actinobacteria numbers [79]. Distinct patterns were revealed in another neurodevelopmental disorder, attention deficit hyperactivity disorder (ADHD). Although ZnO nanoparticles possessed bacteriostatic and bactericidal effects both in healthy children and ADHD patients, ZnO supplementation reduced gut bacteria diversity up to the level observed in healthy controls [80].

In agreement with our earlier suggestion on the contribution of gut dysfunction and microbiota to the role of Zn deficiency in the development of fetal alcohol syndrome [81], a recent study demonstrated that Zn deficiency aggravated alcohol-induced Paneth cell dysfunction with a reduction in α-defensin production, as well as impairment of gut microbiota composition and gut barrier integrity [82].

In obese Korean children, zinc intake was found to be inversely associated with dietary zinc intake [83]. These findings corroborate earlier data on the beneficial role of Bacteroidetes in body weight regulation [84], thus providing an additional potential mechanism for protective effects of Zn in obesity [85].

In contrast to the abovementioned studies, researchers have also demonstrated the potential contribution of intestinal microbiota to Zn neurotoxicity upon overexposure. Specifically, oral exposure to ZnONPs was shown to affect spatial learning, memory, and motor function in mice along with alterations of hippocampal gene expression. Despite the lack of Zn exposure on gut microbiota biodiversity, increased relative abundance of Actinobacteria was observed. At the same time, the observed effects of ZnONPs on serum metabolomics and hippocampal *Bdnf* and *Dlg4* gene expression were found to correlate significantly with ZnONP-induced modulation of gut microflora with *Actinobacteria*, *Bifidobacteria*, *Sutterella*, and *Adlercreutzia* taxa characterized by the most profound association with these variables. Thus, it is plausible that the impact of Zn on brain physiology may be mediated not only through increased gut permeability and elevation of bacterial proinflammatory lipopolysaccharides, but also through modulation of gut microflora metabolites, including the neuroactive ones [86]. Correspondingly, an increased biosynthesis and transport of 5-hydroxytryptamine (5-HT) in gut upon Zn oxide exposure was also shown to result in increased brain 5-HT levels, although the role of gut microflora in this effect is yet to be elucidated [87].

Despite the paucity of studies, the existing data demonstrate that the interplay between Zn and gut microflora not only affects intestinal physiology underlying local effects but may also be involved in the pathogenesis of extraintestinal pathology, including neurological, systemic inflammatory, and metabolic diseases. Moreover, the modulation of gut microbiota may mediate not only the physiological but also the supraphysiological and toxic effects of Zn.

## 5. Probiotics

Given the existing data on the role of Zn in the regulation of gut microbiota, the efficiency of its co-supplementation with probiotics has been investigated in a number of studies.

In heat-exposed Wistar rats, zinc potentiated beneficial effects of probiotics on inflammatory response, heat shock protein levels, and antioxidant enzymes, although the maximal effect was observed in the case of Zn, Se, and probiotic co-supplementation [88]. In another study, a combination of zinc with a probiotic complex and rosavin was shown to ameliorate monosodium iodoacetate-induced osteoarthritis in a rat model through down-regulation of proinflammatory cytokines and catabolic factor expression in cartilage [89].

In turn, a combination of multistrain probiotics with Zn sulfate supplementation significantly improved intestinal morphology in broilers, as revealed by villi height and weight, crypt death, lamina propria thickness, as well as goblet cell number [90]. A similar effect was observed in broilers exposed to heat stress [91]. At the same time, no beneficial effects along with the presence of side-effects like reduced iron absorption and lower hemoglobin levels were observed following the addition of a high Zn sulfate dose to *Lactobacillus reuteri*-based probiotics [92].

The potential beneficial effects of Zn and probiotic co-supplementation may be associated with the mutual interaction of these agents. On the one hand, probiotics were shown to increase Zn bioavailability [93]. Zn also exerts a significant impact on the probiotic microflora, although the effect appears to be highly non-linear. Specifically, Zn at doses of 100—500 mg/l was shown to increase the growth rate of *L. plantarum* CCM 7102, lactate production, and adhesion to enterocytes, as well as inhibit *E. coli* and *S. typhimurium* growth, whereas higher doses reversed these beneficial effects [94]. It is also notable that probiotic supplementation may also counteract certain effects of Zn species like the proinflammatory effect of inorganic ZnSO_4_ in intestinal epithelial cells [95].

Zn may also be involved in the mediation of the protective effects of probiotics. Specifically, probiotic *Escherichia coli* Nissle 1917 (*E. coli* Nissle) was shown to compete with pathogenic *S. typhimurium* for Zn^2+^ due to the presence of Zn-binding siderophore yersiniabactin [96].

Pilot studies have also been performed to evaluate the potential effects of Zn and probiotic supplementation in humans. Early on, Zn was considered a potential tool for the improvement of diarrhea due to its effects on gut permeability, immune system, epithelial function, and electrolyte balance [97], whereas the potential impact of Zn on gut microflora was not considered as protective. However, a recent study demonstrated that Zn may be even more effective in the treatment of diarrhea in children aged 6–24 months as compared to probiotics while also having fewer complications [98]. At the same time, co-supplementation of Zn and microencapsulated *Lactobacillus plantarum* IS-10506 in preschool children did not have added advantages on the effects on fecal IgA levels in comparison to treatment with a probiotic alone; although improved Zn status was proposed to be beneficial for immunity [99]. Probiotic and Zn co-supplementation has also been proposed as a potential tool for the management of hepatic encephalopathy [100].

Generally, the existing data demonstrate the potential usefulness of Zn and probiotic co-supplementation due to certain potentiating effects in animal models of local and systemic inflammation. However, insufficient human data from Zn-probiotic supplementation trials do not conclusively establish the efficiency of the latter.

## 6. Conclusions

**Figure 1:**
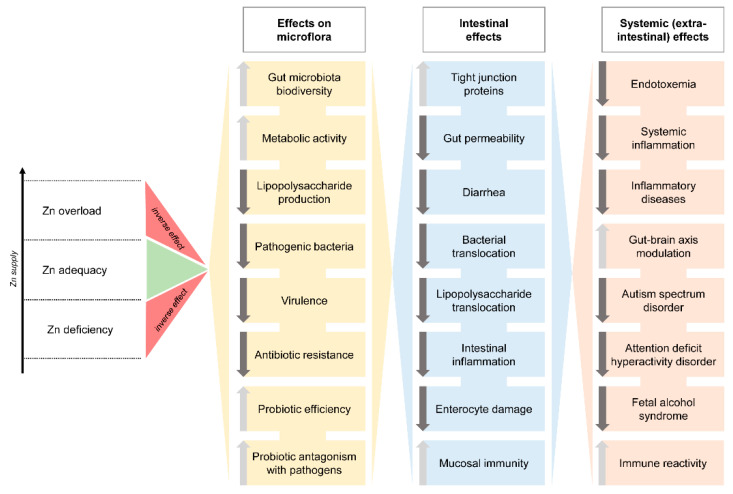
The association between Zn status and gut microbiota in relation to local intestinal and systemic effects.

Despite being rather contradictory and dose- and species-specific, the existing data demonstrate a tight relationship between Zn metabolism and gut microbiota with both Zn deficiency and excess having adverse effects on gut microbiota (Figure 1). Moreover, the interplay between Zn status and intestinal microflora was shown to have significant local and systemic effects. The first one is characterized by improved gut wall integrity and reduced intestinal inflammation. In turn, systemic effects may include systemic inflammation, acute pancreatitis, autism spectrum disorder, attention deficit hyperactivity disorder, fetal alcohol syndrome, and obesity. It is highly likely that further research in the field will unravel additional multilevel effects of Zn mediated by gut microbiota.

In view of both Zn and gut microbiota, as well as their interaction in the regulation of physiological functions of the host organism, addressing these targets through the use of Zn-enriched probiotics may be considered an effective strategy in health management. The advantages of co-supplementation may include increased bioavailability of Zn and improved growth of probiotic bacteria, as well as the beneficial effects of Zn on intestinal microflora and gut integrity. Molecular mechanisms underlying these effects at both host and bacteria levels also require further attention.

## Abbreviations

5-HT—5-hydroxytryptamine; ADHD—attention deficit hyperactivity disorder; ASD—autism spectrum disorder; GFAP—glial fibrillary acidic protein; IFN-γ—interferon γ; IgA—immunoglobulin A; IGF-1—insulin-like growth factor; IL—interleukin; LPS—lipopolysaccharide; MT—metallothionein; NF-κB—nuclear factor κB; NP—nanoparticle; TGF-β1—transforming growth factor β1; TNFα—tumor necrosis factor α; ZnONPs—zinc oxide nanoparticle.

## References

[1] Maret W. (2013). Zinc biochemistry: From a single zinc enzyme to a key element of life. Adv. Nutr..

[2] Skalny A.V., Aschner M., Tinkov A.A. (2021). Zinc. Adv. Food Nutr. Res..

[3] Chasapis C.T., Ntoupa P.A., Spiliopoulou C.A., Stefanidou M.E. (2020). Recent aspects of the effects of zinc on human health. Arch. Toxicol..

[4] Cerasi M., Ammendola S., Battistoni A. (2013). Competition for zinc binding in the host-pathogen interaction. Front. Cell. Infect. Microbiol..

[5] Liuzzi J.P., Lichten L.A., Rivera S., Blanchard R.K., Aydemir T.B., Knutson M.D., Ganz T., Cousins R.J. (2005). Interleukin-6 regulates the zinc transporter Zip14 in liver and contributes to the hypozincemia of the acute-phase response. Proc. Natl. Acad. Sci. USA.

[6] Zackular J.P., Chazin W.J., Skaar E.P. (2015). Nutritional immunity: S100 proteins at the host-pathogen interface. J. Biol. Chem..

[7] Rahman M.T., Karim M.M. (2018). Metallothionein: A Potential Link in the Regulation of Zinc in Nutritional Immunity. Biol. Trace Elem. Res..

[8] Ammendola S., Pasquali P., Pistoia C., Petrucci P., Petrarca P., Rotilio G., Battistoni A. (2007). High-affinity Zn^2+^ uptake system ZnuABC is required for bacterial zinc homeostasis in intracellular environments and contributes to the virulence of Salmonella enterica. Infect. Immun..

[9] McDevitt C.A., Ogunniyi A.D., Valkov E., Lawrence M.C., Kobe B., McEwan A.G., Paton J.C. (2011). A molecular mechanism for bacterial susceptibility to zinc. PLoS Pathog..

[10] Raghupathi K.R., Koodali R.T., Manna A.C. (2011). Size-dependent bacterial growth inhibition and mechanism of antibacterial activity of zinc oxide nanoparticles. Langmuir.

[11] Wu C., Labrie J., Tremblay Y.D., Haine D., Mourez M., Jacques M. (2013). Zinc as an agent for the prevention of biofilm formation by pathogenic bacteria. J. Appl. Microbiol..

[12] Shreiner A.B., Kao J.Y., Young V.B. (2015). The gut microbiome in health and in disease. Curr. Opin. Gastroenterol..

[13] Lee W.J., Hase K. (2014). Gut microbiota-generated metabolites in animal health and disease. Nat. Chem. Biol..

[14] Baquero F., Nombela C. (2012). The microbiome as a human organ. Clin. Microbiol. Infect..

[15] Rooks M.G., Garrett W.S. (2016). Gut microbiota, metabolites and host immunity. Nat. Rev. Immunol..

[16] Clarke G., Stilling R.M., Kennedy P.J., Stanton C., Cryan J.F., Dinan T.G. (2014). Minireview: Gut microbiota: The neglected endocrine organ. Mol. Endocrinol..

[17] Qi X., Yun C., Pang Y., Qiao J. (2021). The impact of the gut microbiota on the reproductive and metabolic endocrine system. Gut Microbes.

[18] Du Toit A. (2019). The gut microbiome and mental health. Nat. Rev. Microbiol..

[19] Southon S., Gee J.M., Bayliss C.E., Wyatt G.M., Horn N., Johnson I.T. (1986). Intestinal microflora, morphology and enzyme activity in zinc-deficient and Zn-supplemented rats. Br. J. Nutr..

[20] Koren O., Tako E. (2020). Chronic Dietary Zinc Deficiency Alters Gut Microbiota Composition and Function. Proceedings.

[21] Shen J., Chen Y., Wang Z., Zhou A., He M., Mao L., Zou H., Peng Q., Xue B., Wang L. (2014). Coated zinc oxide improves intestinal immunity function and regulates microbiota composition in weaned piglets. Br. J. Nutr..

[22] Zhang T., Zhu X., Guo J., Gu A.Z., Li D., Chen J. (2021). Toxicity Assessment of Nano-ZnO Exposure on the Human Intestinal Microbiome, Metabolic Functions, and Resistome Using an In Vitro Colon Simulator. Environ. Sci. Technol..

[23] Durrani M., Nazli R., Sher N., Abubakr M., Ali J. (2021). Gut microbiome profile in zinc deficient infants using next generation sequencing. Khyber Med Univ. J..

[24] Li D., Achkar J.P., Haritunians T., Jacobs J.P., Hui K.Y., D’Amato M., Brand S., Radford-Smith G., Halfvarson J., Niess J.H. (2016). A Pleiotropic Missense Variant in SLC39A8 Is Associated with Crohn’s Disease and Human Gut Microbiome Composition. Gastroenterology.

[25] Collij V., Imhann F., Vich Vila A., Fu J., Dijkstra G., Festen E., Voskuil M.D., Daly M.J., Xavier R.J., Wijmenga C. (2019). SLC39A8 missense variant is associated with Crohn’s disease but does not have a major impact on gut microbiome composition in healthy subjects. PLoS ONE.

[26] Wang Y.H., Zhao W.J., Zheng W.J., Mao L., Lian H.Z., Hu X., Hua Z.C. (2016). Effects of Different Zinc Species on Cellar Zinc Distribution, Cell Cycle, Apoptosis and Viability in MDAMB231 Cells. Biol. Trace Elem. Res..

[27] Reed S., Neuman H., Moscovich S., Glahn R.P., Koren O., Tako E. (2015). Chronic Zinc Deficiency Alters Chick Gut Microbiota Composition and Function. Nutrients.

[28] Reed S., Knez M., Uzan A., Stangoulis J., Glahn R.P., Koren O., Tako E. (2018). Alterations in the Gut (*Gallus gallus*) Microbiota Following the Consumption of Zinc Biofortified Wheat (*Triticum aestivum*)-Based Diet. J. Agric. Food Chem..

[29] Crisol-Martínez E., Stanley D., Geier M.S., Hughes R.J., Moore R.J. (2017). Understanding the mechanisms of zinc bacitracin and avilamycin on animal production: Linking gut microbiota and growth performance in chickens. Appl. Microbiol. Biotechnol..

[30] Nguyen H.T.T., Morgan N., Roberts J.R., Wu S.B., Swick R.A., Toghyani M. (2021). Zinc hydroxychloride supplementation improves tibia bone development and intestinal health of broiler chickens. Poult. Sci..

[31] Fatholahi A., Khalaji S., Hosseini F., Abbasi M. (2021). Nano-Bio zinc synthesized by Bacillus subtilis modulates broiler performance, intestinal morphology and expression of tight junction’s proteins. Livest. Sci..

[32] Gielda L.M., DiRita V.J. (2012). Zinc competition among the intestinal microbiota. MBio.

[33] Rhouma M., Fairbrother J.M., Beaudry F., Letellier A. (2017). Post weanling diarrhea in pigs: Risk factors and non-colistin-based control strategies. Acta Vet. Scand..

[34] Hu C.H., Xiao K., Song J., Luan Z.S. (2013). Effects of zinc oxide supported on zeolite on growth performance, intestinal microflora and permeability, and cytokines expression of weaned pigs. Anim. Feed Sci. Technol..

[35] Wang W., Van Noten N., Degroote J., Romeo A., Vermeir P., Michiels J. (2019). Effect of zinc oxide sources and dosages on gut microbiota and integrity of weaned piglets. J. Anim. Physiol. Anim. Nutr..

[36] Xia T., Lai W., Han M., Han M., Ma X., Zhang L. (2017). Dietary ZnO nanoparticles alters intestinal microbiota and inflammation response in weaned piglets. Oncotarget.

[37] Starke I.C., Pieper R., Neumann K., Zentek J., Vahjen W. (2014). The impact of high dietary zinc oxide on the development of the intestinal microbiota in weaned piglets. FEMS Microbiol. Ecol..

[38] Vahjen W., Pieper R., Zentek J. (2011). Increased dietary zinc oxide changes the bacterial core and enterobacterial composition in the ileum of piglets. J. Anim. Sci..

[39] Liu H., Bai M., Xu K., Zhou J., Zhang X., Yu R., Huang R., Yin Y. (2021). Effects of different concentrations of coated nano zinc oxide material on fecal bacterial composition and intestinal barrier in weaned piglets. J. Sci. Food Agric..

[40] Caine W.R., Metzler-Zebeli B.U., McFall M., Miller B., Ward T.L., Kirkwood R.N., Mosenthin R. (2009). Supplementation of diets for gestating sows with zinc amino acid complex and gastric intubation of suckling pigs with zinc-methionine on mineral status, intestinal morphology and bacterial translocation in lipopolysaccharide-challenged early weaned pigs. Res. Vet. Sci..

[41] Broom L.J., Miller H.M., Kerr K.G., Knapp J.S. (2006). Effects of zinc oxide and Enterococcus faecium SF68 dietary supplementation on the performance, intestinal microbiota and immune status of weaned piglets. Res. Vet. Sci..

[42] Pieper R., Vahjen W., Neumann K., Van Kessel A.G., Zentek J. (2012). Dose-dependent effects of dietary zinc oxide on bacterial communities and metabolic profiles in the ileum of weaned pigs. J. Anim. Physiol. Anim. Nutr..

[43] Wang L., Urriola P.E., Luo Z.H., Rambo Z.J., Wilson M.E., Torrison J.L., Shurson G.C., Chen C. (2016). Metabolomics revealed diurnal heat stress and zinc supplementation-induced changes in amino acid, lipid, and microbial metabolism. Physiol. Rep..

[44] Sauer A.K., Grabrucker A.M. (2019). Zinc Deficiency during Pregnancy Leads to Altered Microbiome and Elevated Inflammatory Markers in Mice. Front. Neurosci..

[45] Kable M.E., Riazati N., Kirschke C.P., Zhao J., Tepaamorndech S., Huang L. (2020). The Znt7-null mutation has sex dependent effects on the gut microbiota and goblet cell population in the mouse colon. PLoS ONE.

[46] Mayneris-Perxachs J., Bolick D.T., Leng J., Medlock G.L., Kolling G.L., Papin J.A., Swann J.R., Guerrant R.L. (2016). Protein- and zinc-deficient diets modulate the murine microbiome and metabolic phenotype. Am. J. Clin. Nutr..

[47] Foligné B., George F., Standaert A., Garat A., Poiret S., Peucelle V., Ferreira S., Sobry H., Muharram G., Lucau-Danila A. (2020). High-dose dietary supplementation with zinc prevents gut inflammation: Investigation of the role of metallothioneins and beyond by transcriptomic and metagenomic studies. FASEB J..

[48] Davis E., Wong C., Bouranis J., Sharpton T., Ho E. (2020). Zinc Status Elicits Age-Dependent Effects in the Gut Microbiome. Curr. Dev. Nutr..

[49] Plum L.M., Rink L., Haase H. (2010). The essential toxin: Impact of zinc on human health. Int. J. Environ. Res. Public Health.

[50] Podany A., Rauchut J., Wu T., Kawasawa Y.I., Wright J., Lamendella R., Soybel D.I., Kelleher S.L. (2019). Excess Dietary Zinc Intake in Neonatal Mice Causes Oxidative Stress and Alters Intestinal Host-Microbe Interactions. Mol. Nutr. Food Res..

[51] Wang C., Cheng K., Zhou L., He J., Zheng X., Zhang L., Zhong X., Wang T. (2017). Evaluation of Long-Term Toxicity of Oral Zinc Oxide Nanoparticles and Zinc Sulfate in Mice. Biol. Trace Elem. Res..

[52] Yoo A., Lin M., Mustapha A. (2021). Zinc Oxide and Silver Nanoparticle Effects on Intestinal Bacteria. Materials.

[53] Ishaq S.L., Page C.M., Yeoman C.J., Murphy T.W., Van Emon M.L., Stewart W.C. (2019). Zinc AA supplementation alters yearling ram rumen bacterial communities but zinc sulfate supplementation does not. J. Anim. Sci..

[54] Oh H.-J., Park Y.-J., Cho J.H., Song M.-H., Gu B.-H., Yun W., Lee J.-H., An J.-S., Kim Y.-J., Lee J.-S. (2021). Changes in Diarrhea Score, Nutrient Digestibility, Zinc Utilization, Intestinal Immune Profiles, and Fecal Microbiome in Weaned Piglets by Different Forms of Zinc. Animals.

[55] Wu R., Mei X., Wang J., Sun W., Xue T., Lin C., Xu D. (2019). Zn(ii)-Curcumin supplementation alleviates gut dysbiosis and zinc dyshomeostasis during doxorubicin-induced cardiotoxicity in rats. Food Funct..

[56] Wu R., Mei X., Ye Y., Xue T., Wang J., Sun W., Lin C., Xue R., Zhang J., Xu D. (2019). Zn(II)-curcumin solid dispersion impairs hepatocellular carcinoma growth and enhances chemotherapy by modulating gut microbiota-mediated zinc homeostasis. Pharmacol. Res..

[57] Gaulke C.A., Rolshoven J., Wong C.P., Hudson L.G., Ho E., Sharpton T.J. (2018). Marginal Zinc Deficiency and Environmentally Relevant Concentrations of Arsenic Elicit Combined Effects on the Gut Microbiome. mSphere.

[58] Sheng Y., Fan F., Jensen O., Zhong Z., Kan B., Wang H., Zhu J. (2015). Dual Zinc Transporter Systems in Vibrio cholerae Promote Competitive Advantages over Gut Microbiome. Infect. Immun..

[59] Shao Y., Lei Z., Yuan J., Yang Y., Guo Y., Zhang B. (2014). Effect of zinc on growth performance, gut morphometry, and cecal microbial community in broilers challenged with Salmonella enterica serovar typhimurium. J. Microbiol..

[60] Sarkar P., Saha T., Sheikh I.A., Chakraborty S., Aoun J., Chakrabarti M.K., Rajendran V.M., Ameen N.A., Dutta S., Hoque K.M. (2019). Zinc ameliorates intestinal barrier dysfunctions in shigellosis by reinstating claudin-2 and -4 on the membranes. Am. J. Physiol. Gastrointest. Liver Physiol..

[61] Zackular J.P., Skaar E.P. (2018). The role of zinc and nutritional immunity in Clostridium difficile infection. Gut Microbes.

[62] Zackular J.P., Moore J.L., Jordan A.T., Juttukonda L.J., Noto M.J., Nicholson M.R., Crews J.D., Semler M.W., Zhang Y., Ware L.B. (2016). Dietary zinc alters the microbiota and decreases resistance to Clostridium difficile infection. Nat. Med..

[63] Parvataneni S., Dasari A.R. (2020). Zinc Level and Its Role in Recurrent Clostridium difficile Infection: A Case Report and Literature Review. J. Investig. Med. High Impact Case Rep..

[64] Feng D., Zhang M., Tian S., Wang J., Zhu W. (2020). Chitosan-chelated zinc modulates cecal microbiota and attenuates inflammatory response in weaned rats challenged with *Escherichia coli*. J. Microbiol..

[65] Pei X., Xiao Z., Liu L., Wang G., Tao W., Wang M., Zou J., Leng D. (2019). Effects of dietary zinc oxide nanoparticles supplementation on growth performance, zinc status, intestinal morphology, microflora population, and immune response in weaned pigs. J. Sci. Food Agric..

[66] Johanns V.C., Epping L., Semmler T., Ghazisaeedi F., Lübke-Becker A., Pfeifer Y., Eichhorn I., Merle R., Bethe A., Walther B. (2020). High-Zinc Supplementation of Weaned Piglets Affects Frequencies of Virulence and Bacteriocin Associated Genes Among Intestinal *Escherichia coli* Populations. Front. Vet. Sci..

[67] Crane J.K., Naeher T.M., Shulgina I., Zhu C., Boedeker E.C. (2007). Effect of zinc in enteropathogenic *Escherichia coli* infection. Infect. Immun..

[68] Bücker R., Zakrzewski S.S., Wiegand S., Pieper R., Fromm A., Fromm M., Günzel D., Schulzke J.D. (2020). Zinc prevents intestinal epithelial barrier dysfunction induced by alpha-hemolysin-producing *Escherichia coli* 536 infection in porcine colon. Vet. Microbiol..

[69] Chen L., Wang Z., Wang P., Yu X., Ding H., Wang Z., Feng J. (2021). Effect of Long-Term and Short-Term Imbalanced Zn Manipulation on Gut Microbiota and Screening for Microbial Markers Sensitive to Zinc Status. Microbiol. Spectr..

[70] Ciesinski L., Guenther S., Pieper R., Kalisch M., Bednorz C., Wieler L.H. (2018). High dietary zinc feeding promotes persistence of multi-resistant *E. coli* in the swine gut. PLoS ONE.

[71] Gammoh N.Z., Rink L. (2017). Zinc in Infection and Inflammation. Nutrients.

[72] Souffriau J., Timmermans S., Vanderhaeghen T., Wallaeys C., Van Looveren K., Aelbrecht L., Dewaele S., Vandewalle J., Goossens E., Verbanck S. (2020). Zinc inhibits lethal inflammatory shock by preventing microbe-induced interferon signature in intestinal epithelium. EMBO Mol. Med..

[73] Straub R.H., Schradin C. (2016). Chronic inflammatory systemic diseases: An evolutionary trade-off between acutely beneficial but chronically harmful programs. Evol. Med. Public Health.

[74] Su S.Y., Tang Q.Q. (2021). Altered intestinal microflora and barrier injury in severe acute pancreatitis can be changed by zinc. Int. J. Med. Sci..

[75] Lee A.A., Baker J.R., Wamsteker E.J., Saad R., DiMagno M.J. (2019). Small Intestinal Bacterial Overgrowth Is Common in Chronic Pancreatitis and Associates With Diabetes, Chronic Pancreatitis Severity, Low Zinc Levels, and Opiate Use. Am. J. Gastroenterol..

[76] Gordon S.R., Vaishnava S. (2019). Zinc supplementation modulates T helper 17 cells via the gut microbiome. J. Immunol..

[77] Vela G., Stark P., Socha M., Sauer A.K., Hagmeyer S., Grabrucker A.M. (2015). Zinc in gut-brain interaction in autism and neurological disorders. Neural. Plast..

[78] Wong G.C., Jung Y., Lee K., Fourie C., Handley K.M., Montgomery J.M., Taylor M.W. (2021). Effect of dietary zinc supplementation on the gastrointestinal microbiota and host gene expression in the Shank3B-/-mouse model of autism spectrum disorder. bioRxiv.

[79] Yu R., Ahmed T., Jiang H., Zhou G., Zhang M., Lv L., Li B. (2021). Impact of Zinc Oxide Nanoparticles on the Composition of Gut Microbiota in Healthy and Autism Spectrum Disorder Children. Materials.

[80] Zhou G., Yu R., Ahmed T., Jiang H., Zhang M., Lv L., Alhumaydhi F.A., Allemailem K.S., Li B. (2021). Biosynthesis and Characterization of Zinc Oxide Nanoparticles and Their Impact on the Composition of Gut Microbiota in Healthy and Attention-Deficit Hyperactivity Disorder Children. Front. Microbiol..

[81] Skalny A.V., Skalnaya M.G., Grabeklis A.R., Skalnaya A.A., Tinkov A.A. (2018). Zinc deficiency as a mediator of toxic effects of alcohol abuse. Eur. J. Nutr..

[82] Zhong W., Wei X., Hao L., Lin T.D., Yue R., Sun X., Guo W., Dong H., Li T., Ahmadi A.R. (2020). Paneth Cell Dysfunction Mediates Alcohol-related Steatohepatitis through Promoting Bacterial Translocation in Mice: Role of Zinc Deficiency. Hepatology.

[83] Shin S., Cho K.Y. (2020). Altered Gut Microbiota and Shift in Bacteroidetes between Young Obese and Normal-Weight Korean Children: A Cross-Sectional Observational Study. Biomed. Res. Int..

[84] Crovesy L., Masterson D., Rosado E.L. (2020). Profile of the gut microbiota of adults with obesity: A systematic review. Eur. J. Clin. Nutr..

[85] Olechnowicz J., Tinkov A., Skalny A., Suliburska J. (2018). Zinc status is associated with inflammation, oxidative stress, lipid, and glucose metabolism. J. Physiol. Sci..

[86] Chen J., Zhang S., Chen C., Jiang X., Qiu J., Qiu Y., Zhang Y., Wang T., Qin X., Zou Z. (2020). Crosstalk of gut microbiota and serum/hippocampus metabolites in neurobehavioral impairments induced by zinc oxide nanoparticles. Nanoscale.

[87] Zhang S., Cheng S., Jiang X., Zhang J., Bai L., Qin X., Zou Z., Chen C. (2020). Gut-brain communication in hyperfunction of 5-hydroxytryptamine induced by oral zinc oxide nanoparticles exposure in young mice. Food Chem. Toxicol..

[88] Malyar R.M., Li H., Liu D., Abdulrahim Y., Farid R.A., Gan F., Ali W., Enayatullah H., Banuree S., Huang K. (2020). Selenium/Zinc-Enriched probiotics improve serum enzyme activity, antioxidant ability, inflammatory factors and related gene expression of Wistar rats inflated under heat stress. Life Sci..

[89] Kwon J.Y., Lee S.H., Jhun J., Choi J., Jung K., Cho K.H., Kim S.J., Yang C.W., Park S.H., Cho M.L. (2018). The Combination of Probiotic Complex, Rosavin, and Zinc Improves Pain and Cartilage Destruction in an Osteoarthritis Rat Model. J. Med. Food.

[90] Shah M., Zaneb H., Masood S., Khan R.U., Ashraf S., Sikandar A., Rehman H., Rehman H.U. (2019). Effect of Dietary Supplementation of Zinc and Multi-Microbe Probiotic on Growth Traits and Alteration of Intestinal Architecture in Broiler. Probiotics Antimicrob Proteins.

[91] Shah M., Zaneb H., Masood S., Khan R.U., Mobashar M., Khan I., Din S., Khan M.S., Rehman H.U., Tinelli A. (2020). Single or Combined Applications of Zinc and Multi-strain Probiotic on Intestinal Histomorphology of Broilers Under Cyclic Heat Stress. Probiotics Antimicrob Proteins.

[92] Kelleher S.L., Casas I., Carbajal N., Lönnerdal B. (2002). Supplementation of infant formula with the probiotic lactobacillus reuteri and zinc: Impact on enteric infection and nutrition in infant rhesus monkeys. J. Pediatr. Gastroenterol. Nutr..

[93] Bergillos-Meca T., Navarro-Alarcón M., Cabrera-Vique C., Artacho R., Olalla M., Giménez R., Moreno-Montoro M., Ruiz-Bravo A., Lasserrot A., Ruiz-López M.D. (2013). The probiotic bacterial strain Lactobacillus fermentum D3 increases in vitro the bioavailability of Ca, P, and Zn in fermented goat milk. Biol. Trace Elem. Res..

[94] Mudroňová D., Gancarčíková S., Nemcová R. (2019). Influence of Zinc Sulphate on the Probiotic Properties of Lactobacillus plantarum CCM 7102. Folia Vet..

[95] Šefcová M., Levkut M., Bobíková K., Karaffová V., Revajová V., Maruščáková I.C., Levkutová M., Ševčíková Z., Herich R., Levkut M. (2019). Cytokine response after stimulation of culture cells by zinc and probiotic strain. Vitr. Cell. Dev. Biol. Anim..

[96] Behnsen J., Liu J., Valeri M., Hoover E., Tjokrosurjo J., Montaldo N.P., Treacy-Abarca S., Garibay O., Gilston B.A., Edwards R.A. (2017). Probiotic *Escherichia coli* Nissle 1917 Uses Zinc Transporters and the Siderophore Yersiniabactin to Acquire Zinc in the Inflamed Gut and Outcompete Salmonella Typhimurium. FASEB J..

[97] Salvatore S., Hauser B., Devreker T., Vieira M.C., Luini C., Arrigo S., Nespoli L., Vandenplas Y. (2007). Probiotics and zinc in acute infectious gastroenteritis in children: Are they effective?. Nutrition.

[98] Ahmadipour S., Mohsenzadeh A., Alimadadi H., Salehnia M., Fallahi A. (2019). Treating Viral Diarrhea in Children by Probiotic and Zinc Supplements. Pediatr. Gastroenterol. Hepatol. Nutr..

[99] Surono I.S., Martono P.D., Kameo S., Suradji E.W., Koyama H. (2014). Effect of probiotic L. plantarum IS-10506 and zinc supplementation on humoral immune response and zinc status of Indonesian pre-school children. J. Trace Elem. Med. Biol..

[100] Gonzales A.D., Reinert J.P. (2020). Zinc and Probiotic Therapy for Management of Hepatic Encephalopathy. Sr. Care Pharm..

